# Caregivers’ Malaria Knowledge, Beliefs and Attitudes, and Related Factors in the Bata District, Equatorial Guinea

**DOI:** 10.1371/journal.pone.0168668

**Published:** 2016-12-30

**Authors:** Maria Romay-Barja, Policarpo Ncogo, Gloria Nseng, Maria A. Santana-Morales, Zaida Herrador, Pedro Berzosa, Basilio Valladares, Matilde Riloha, Agustin Benito

**Affiliations:** 1 Centro Nacional de Medicina Tropical, Instituto de Salud Carlos III, Madrid, Spain; 2 Red de Investigación Colaborativa en Enfermedades Tropicales, RICET, Madrid, Spain; 3 Centro de Referencia de Control de Endemias, Malabo, Equatorial Guinea; 4 Ministerio de Salud y Bienestar Social, Malabo, Equatorial Guinea; 5 Instituto Universitario de Enfermedades Tropicales y Salud Pública de Canarias, Universidad de La Laguna, Tenerife, Spain; Agencia de Salut Publica de Barcelona, SPAIN

## Abstract

**Objectives:**

Adequate community knowledge about malaria is crucial in order to improve prevention by reducing exposure to the disease. Malaria is a major cause of morbidity and mortality among children of less than five years of age in Equatorial Guinea. However, information concerning the accuracy of community knowledge is insufficient. This study aimed at assessing the depth of caregivers’ knowledge of malaria, their beliefs and attitudes about this disease, and their socioeconomic determinants in the Bata district of Equatorial Guinea.

**Methodology:**

A cross-sectional study was conducted in the district of Bata, involving 440 houses selected from 18 rural villages and 26 urban neighbourhoods. A combined "Malaria Knowledge Score" was generated based on caregivers’ knowledge about transmission, symptoms, prevention, the treatment of children, and best place to seek treatment. Multivariate logistic regressions analyses were performed to assess those factors that are associated with knowledge about malaria.

**Results:**

A total of 428 caregivers were interviewed; 255 (59.6%) and 173 (40.4%) lived in urban and rural areas respectively. Significant differences between rural and urban households were observed in caregivers’ malaria knowledges and beliefs. Almost 42% of urban and 65% of rural caregivers were unaware as to how malaria is transmitted (OR = 2.69; 95% CI: 1.78–4.05). Together with rurality, the factors most significantly associated with the Malaria Knowledge were the level of education of the caregiver and the socioeconomic status of the household.

**Conclusions:**

Improvements in educational programs are needed to empower the most vulnerable households such that they can pro-actively implement malaria control measures. This could be achieved by a comprehensive communication strategy aimed at changing individual and community behaviours, and delivered by suitably trained community health workers and indoor residual spraying personnel.

## Introduction

The increasing investments in malaria have contributed to a substantial decrease in incidence and mortality during the last decade even though the disease remains a major public health problem in Africa. There are over 200 million cases of malaria each year, most of which (88%) occur in children under five years of age in the sub-Saharan region [1].

Disease prevention is of prime importance in reducing the rates of morbidity and mortality, but its everyday practice in households is related to local perceptions of risk and knowledge about malaria [2,3]. Adequate community knowledge about malaria is crucial to guarantee that preventative measures are correctly applied and disease exposure reduced [4]. However, misconceptions about malaria frequently persist among communities and individual households that may jeopardise the success of disease prevention [5,6].

Several characteristics distinguish urban from rural malaria [7]. Rural and urban populations differ in their cultural practices, socioeconomic and demographic characteristics, availability and accessibility to health services. Urban populations are generally younger and better educated than rural populations [8]. Rural areas are linked with increased levels of poverty together with diminished access to healthcare facilities due to their remoteness [9]. -Both rural and urban populations should be taken into account to improve the efficacy and efficiency of control interventions.

There have been a considerable number of studies about malaria knowledge, attitudes and practices in Africa concluding that the knowledge about malaria is related with various socio-economic determinants [10,11]. The level of household knowledge about malaria and its determinants, especially in endemic countries, should be considered for successful implementation of malaria control programmes [12].

Improvements in caregivers’ health education can reduce significantly under-five malaria morbidity and mortality [13]. Communication activities aimed at generating behavioural changes, based on face-to face education strategies, can foster positive changes in attitude to disease control compared to impersonal mass media campaigns [14]. In the assessment of the potential ways of improving malaria control strategies, there is global agreement on the necessity of operational research that would help to create context specific messages based in scientific evidence [15].

In Equatorial Guinea, malaria is endemic, with stable transmission and remains a major cause of morbidity and mortality among children under five years of age [16,17]. A malaria control programme was introduced in 2007 in the mainland region, under the Equatorial Guinea Malaria Control Initiative (EGMCI). The strategy consisted, mainly, of indoor spraying in the Litoral and Kie-Ntem provinces, as well as the mass distribution of nets treated with long-lasting insecticide in the Centro Sur and Wele-Nzas provinces. Case management was improved through the distribution of free artemisinin-based combination therapy and other measures. An integrated set of information, education, and communications activities were promulgated [18]. The initiative was largely funded by The Global Fund to Fight AIDS, Tuberculosis, and Malaria (GFATM), and it was implemented by the government of Equatorial Guinea, in collaboration with several international organizations. The first-line treatment for uncomplicated malaria in Equatorial Guinea is artesunate–amodiaquine (AS + AQ) while quinine is recommended for severe malaria cases. Unfortunately, with the withdrawal of the GFATM funding in 2011, the EGMCI program stopped its main activities and free universal access to ACT was not sustained in mainland region of Equatorial Guinea, including the Bata District [18,19]. Despite the efforts made by the EGMCI program, the prevalence of malaria has remained high (41.2%) in the Bata district in children under 15 years of age [20].

Information regarding community knowledge concerning malaria in Equatorial Guinea is insufficient. A more-detailed analysis may be helpful to develop appropriate educational and communication strategies aimed to increase community disease prevention and control. The purpose of this study was to assess the caregivers’ knowledge of malaria, beliefs, and attitudes, and related socioeconomic factors in the Bata district of Equatorial Guinea.

## Materials and Methods

### The study area and survey

The District of Bata, with its population of 244,264 inhabitants, is the largest in the country according to the latest national census [21]. As described in detail previously [22], the District’s public health facilities comprise a network of ten health centres, two rural and eight urban, and one regional hospital located in the city of Bata. There are also private health facilities in the Bata District, including two hospitals, and about 23 clinics, all in the urban area of Bata city. Therefore, government health services, private practitioners and pharmacies are concentrated in urban areas (S1 Fig). In rural areas, where the majority of the population lives further than 3 km from a health facility, 99 Community Health Workers (CHWs) covering 68 rural communities were trained to manage malaria diagnosis and treatment but only 35 are currently working.

This cross-sectional study was carried out from June–August 2013, in the Bata district of mainland Equatorial Guinea. The study was part of a project that aimed to provide baseline data on the prevalence of malaria, the genetic characteristics of *Plasmodium falciparum*, vectors involved in the transmission, and to provide base-line information about the malaria-related knowledge, attitudes and practices of households related to malaria episodes for children 15 years and under.

### Study population, sampling, and data collection

A descriptive cross-sectional study was designed to determine the knowledge, attitudes and practices of rural and urban households related to malaria in the District of Bata. Sampling was carried out with a multistage stratified cluster strategy. First, 18 rural villages, out of 70, and 26 urban neighbourhoods, out of 111, were randomly selected with probability proportional to size to better assure representativeness in the sample design. Then, 440 households were randomly selected from an updated census from each cluster provided by the head of the village or neighbourhood. The particulars of the survey have been described previously in detail [19,20].

Household caregivers were identified in each house with at least one child under 15 years of age, and asked about their knowledge, beliefs, and attitudes related to malaria. An open-ended questionnaire was designed and administered (unprompted) by trained field workers. The questionnaire was previously tested and translated into the main local language, Fang. Information regarding household social characteristics was also recorded.

### Data analyses

A wealth indicator variable that served as a proxy for socio-economic status was created with household-owned assets, housing characteristics and the type of access to water and sanitation using principal component analysis [18,19]. The first principal component was considered as the summary measure of socio-economic status, and subsequently divided into quintiles to assign households to different wealth strata.

A descriptive analysis of participants’ and household characteristics was carried out using frequency tables for categorical variables and mean and standard deviation or median and interquartile range for normally and not-normally distributed continuous variables, respectively. Differences in knowledge, beliefs and attitudes between rural and urban caregivers were assessed using the chi-squared test and the Student-t test for categorical and continuous variables, respectively. Comparisons for which p values were below 0.05 were considered significant. Odds ratio (OR) and confidence interval (CI) were estimated by using logistic regression.

In order to assess the effect of socio-economic variables on caregivers’ knowledge of malaria, a Knowledge Score was created allowing for categorizing respondents as low or high knowledge. Following the commonest method of calculating combined scores and categorizing them from quantitative knowledge [2,23–26], a composite malaria knowledge score was created for each caregiver where every correct answer received a single point. These included caregivers’ correct knowledge on malarial transmission, disease symptoms, prevention, treatment for children and treatment centres. To achieve a maximum score the respondents had to know that malaria is transmitted by a mosquito (1 point); can be lethal (1 point); is more dangerous in children (1 point); could cause symptoms such as fever (1 point), as well as headache, body pains, or convulsions (1 point). The respondent also needed to know that bed net could be used to prevent malaria (1 point), as well as indoor spraying (1 point) and other preventative measures (1 point). Also, the respondents had to know that the stagnant water is a mosquito breeding site (1 point) as well as to know the correct malaria mosquito biting time (1 point). AS/AQ or Quinine had to be mentioned as malaria treatment for a child (1 point each) and a health facility as the place to seek treatment for a child with malaria (1 point). Altogether, the highest possible score was 13 points. High and Low Knowledge of Malaria were defined as scores either above or within and below the overall median, respectively [27].

A bivariate analysis of the associations between the independent socioeconomic variables and the Knowledge of Malaria score was performed using logistic regression. Collinearity between independent variables was checked and in case of collinearity, the variable explaining less of data distribution was removed. The multivariable logistic regression model used to determine factors associated with a high Knowledge of Malaria score was then adjusted by area: urban or rural. The odds ratio (OR) and 95% confidence interval (95% CI) were computed; p values less than or equal to 0.05 were considered statistically significant. The design effect associated with the sampling strategy applied for this study was estimated in the analysis using the Stata function “svyset” to declare the survey design. Data analyses were performed using STATA software version 12.

### Ethics statement

This study was approved by the Ministry of Health and Social Welfare of Equatorial Guinea and the Ethics Committee of the Spanish National Health Institute, Carlos III (CEI PI 22_2013-v3). Written informed consent for participation in this study was obtained from the caregivers interviewed, and from the heads of household.

## Results

### Socio-demographic characteristics

A total of 428 caregivers were interviewed about their malaria knowledge, beliefs and attitudes out of which 255 (59.58%) lived in urban area (Table 1). Caregivers were younger in urban than in rural areas, with a median age of 30 (IQR: 25–40; minimum:15; maximum: 66) and 40 (IQR: 30–50; minimum:15; maximum: 70) years respectively. In urban Bata, 63.92% of caregivers had attained secondary school or more, whereas this percentage fell to 36.99% in rural area (p = <0.001). The presence of a malaria case was 1.25 times more frequent in rural than in urban Bata, where 78.91% of the households had experienced at least one case of malaria at the time of the survey compared to 62.15% households in urban areas.

**Table 1:** Characteristics of caregivers and households at Bata district, by area.

	Urban (n = 255)		Rural (n = 173)		
	n	%	n	%	P-value
**Sex**		** **	** **	** **	** **
Male	4	1.57	3	1.73	
Female	251	98.43	170	98.27	0.895
**Age**					
15–24	63	24.71	23	13.29	
25–34	108	42.35	45	26.01	
35–44	47	18.43	35	20.23	
45–54	24	9.41	47	27.17	
55 & +	13	5.10	23	13.29	<0.001
**Education**					
Primary school or less	92	36.08	109	63.01	
Secondary school or more	163	63.92	64	36.99	<0.001
Ethnicity					
Fang	217	85.10	150	86.71	
Combe	20	7.84	11	6.36	
Bisio	2	0.78	4	2.31	
Ndowe	16	6.27	8	4.62	0.464
**Wealth**					
Poorest	10	3.92	77	44.51	
Second	40	15.69	50	28.90	
Middle	59	23.14	21	12.14	
Fourth	71	27.84	16	9.25	
Richest	75	29.41	9	5.20	<0.001
**Malaria case in the house**					
No	95	37.85	31	21.09	
Yes	156	62.15	116	78.91	0.001

### Malaria awareness and transmission knowledge

Asked about the principal health problem in their community or neighbourhood (Table 2), malaria was the primary issue for 87.86% of rural respondents versus 77.25% of urban (p = 0.006).

**Table 2:** Caregivers’ knowledge of malaria disease symptoms and transmission in Bata district by area.

	Urban (n = 255)		Rural (n = 173)				
	n	%	n	%	P-value	OR	(95% CI)
**What is the principal health problem in your community?**							
Other diseases	58	22.75	21	12.14			
Malaria	197	77.25	152	87.86	0.006	2.13	(1.23–3.69)
**Who suffer most frequently from malaria?**							
Other family members	75	29.41	60	34.68			
Children	180	70.59	113	65.32	0.250	0.78	(0.52–1.19)
**To whom could malaria be more dangerous?**							
Others	32	12.55	36	20.81			
Children	223	87.45	137	79.19	0.022	0.55	(0.32–0.92)
**Can malaria be lethal?**							
No	16	6.27	11	6.36			
Yes	239	93.73	162	93.64	0.972	0.99	(0.45–2.18)
**Main malaria symptoms in children**							
Fever	214	83.92	111	64.16	<0.001	0.34	(0.21–0.55)
Nausea	58	22.75	29	16.76	0.131	0.68	(0.42–1.12)
Headache	34	13.33	35	20.23	0.057	1.65	(0.98–2.77)
Convulsions	15	5.88	36	20.81	<0.001	4.20	(2.18–8.10)
Weakness	34	13.33	14	8.09	0.092	0.58	(0.30–1.10)
Body pain	9	3.53	11	6.36	0.174	1.86	(0.75–0.59)
**How is malaria transmitted?**							
Mosquito bite	148	58.04	61	35.26	<0.001	0.39	(0.26–0.59)
Don't know	92	36.08	102	58.96	<0.001	2.69	(1.78–4.05)
Others	15	5.88	10	5.78	0.266	1.62	(0.69–3.81)

Regarding specific topics of malaria competence, 79.19% of rural (versus 87.45% of urban) caregivers believed that malaria is more dangerous in children (p = 0.022). Caregivers in rural households had one-third the odds of recognizing fever as a malarial symptom than those from urban households. Convulsions was the second most common symptom (20.85%) mentioned in rural areas, with nausea being the second most common symptom mentioned (22.75%) in urban households. Additionally, 64.74% of rural caregivers were unaware of malaria transmission patterns versus 41.96% of urban caregivers, and only 35.26% of rural households (versus 58.04% of urban) knew that a mosquito bite is the means of transmission.

### Knowledge of mosquito habits and malaria prevention

Malaria caregivers were asked separately about the best way to prevent malaria and the best way to avoid mosquito bites (Table 3). Regarding the first, 54.12% of urban caregivers and 33.53% of rural answered bed nets as the best preventative measure (OR: 0.43 95% C.I. 0.28–0.64). Some misconceptions, like believing that boiling drinking water could prevent malaria are still present in Bata district, even in urban caregivers. Regarding the best way to avoid mosquito bites, most caregivers mentioned bed nets (71.76%), with no significant differences between areas. The spraying of insecticide was significantly less mentioned by rural caregivers (OR: 0.44 95%CI: 0.24–0.81). However, most caregivers answered that they would allow government workers to spray their house, with no significant differences between areas (89.02% urban and 91.91% rural).

**Table 3:** Caregivers’ knowledge about malaria prevention and mosquito habits in Bata district by area.

	Urban (n = 255)		Rural (n = 173)				
	n	%	n	%	P-value	OR	(95% CI)
**Malaria is preventable**	187	73.33	108	62.43	0.017	0.60	(0.40–0.91)
**Best way to prevent malaria**							
Sleep under a bed net	138	54.12	58	33.53	<0.001	0.43	(0.28–0.64)
Clean the house and environs	33	12.94	24	13.87	0.781	1.08	(0.62–1.91)
Take preventive medication	26	10.20	23	13.29	0.323	1.35	(0.74–2.46)
Indoor spraying	21	8.24	5	2.89	0.023	0.33	(0.12–0.90)
Boil drinking water	17	6.67	4	2.31	0.041	0.33	(0.11–1.01)
Drain/cover stagnant water	8	3.14	8	4.62	0.426	1.50	(0.55–4.08)
Generate smoke	1	0.39	1	0.58	0.781	1.48	(0.09–23.85)
Do not walk barefoot	1	0.39	5	2.89	0.031	7.56	(0.86–66.22)
**Best way to avoid a mosquito bite**							
Sleep under a bed net	183	71.76	125	72.25	0.912	1.02	(0.67–1.58)
Indoor spraying	47	18.43	16	9.25	0.006	0.44	(0.24–0.81)
Use mosquito coil	27	10.59	10	5.78	0.082	0.52	(0.24–1.10)
Remain fully clothed	3	1.18	7	4.05	0.054	3.54	(0.90–14.00)
Clean the house and environs	1	0.39	6	3.47	0.014	9.13	(1.07–77.85)
Generate smoke	1	0.39	2	1.16	0.353	2.97	(0.27–33.19)
Nets in the windows	1	0.39	4	2.31	0.070	6.01	(0.66–54.86)
Keep the door of the house closed	4	1.57	1	0.58	0.349	0.36	(0.04–3.31)
**Breeding sites of mosquitoes**							
Garbage	60	23.53	46	26.59	0.472	1.18	(0.75–1.84)
Everywhere	48	18.82	30	17.34	0.697	0.90	(0.55–1.50)
Puddles	47	18.43	32	18.50	0.986	1.00	(0.61–1.65)
Stagnant Water	33	12.94	8	4.62	0.004	0.33	(0.15–0.73)
Grass	23	9.02	17	9.83	0.778	1.10	(0.57–2.13)
Dark places	14	5.49	5	2.89	0.200	0.51	(0.18–1.45)
Latrines	5	1.96	13	7.51	0.005	4.06	(1.41–11.74)
Don't know	25	9.80	22	12.72	0.344	1.34	(0.73–2.47)
**Biting time of mosquitoes that transmit malaria**							
Dawn	1	0.39	2	1.16	0.353	2.97	(0.27–33.19)
Morning	3	1.18	5	2.89	0.199	2.50	(0.59–10.65)
Evening	3	1.18	7	4.05	0.054	3.54	(0.90–14.00)
Dusk	19	7.45	22	12.72	0.069	1.81	(0.94–3.47)
Night time	217	85.10	124	71.68	0.001	0.44	(0.27–0.72)
Both day and night time	9	3.53	9	5.20	0.397	1.50	(0.58–3.87)
Don't know	3	1.18	4	2.31	0.363	1.99	(0.44–9.03)
**Correct biting time**							
No	18	7.06	25	14.45			
Yes	237	92.94	148	85.55	0.013	0.45	(0.24–0.86)

Related to mosquito habits, 24.77% of the caregivers answered that garbage could serve as breeding site for mosquitoes. Also, 18.46% of caregivers in both areas said that puddles could serve as breeding site. Only 12.94% of urban caregivers, and 4.62% of rural, knew that stagnant water is the breeding place for mosquitoes. Regarding the time when malaria mosquitoes are most likely to bite, most caregivers (79.67%) answered that night-time was the riskiest, while rural households were less likely to give this answer than urban ones (OR: 0.45 95% CI: 0.24–0.86).

Table 4 contains the caregivers’ knowledge about malaria treatment, treatment seeking-behaviour and their sources of information. Asked about where they would go to seek treatment for a child with malaria, caregivers from both areas answered hospital, mainly, although this answer was significantly less mentioned from rural caregivers (OR: 0.56 95%CI:0.37–0.87). Caregivers in rural areas had 2.36 times the odds of mentioning Health Centres compared to urban areas. Regarding optimal medicines for use with children with malaria, the commonest antimalarial mentioned was Artemether (mentioned by 32.16% of urban respondents versus 16.18% of rural), followed by AS+AQ.

**Table 4:** Best treatment, best place to seek treatment for a child with malaria and sources of information by area.

	Urban (n = 255)		Rural (n = 173)				
	n	%	n	%	P-value	OR	(95% CI)
**Best treatment for a child with malaria**							
Artemether	82	32.16	28	16.18	<0.001	0.41	(0.25–0.67)
Paracetamol	70	27.45	52	30.06	0.558		
Fansidar	53	20.78	19	10.98	0.008	0.47	(0.27–0.83)
AS/AQ	50	19.61	21	12.14	0.041	0.57	(0.33–0.99)
Quinine	37	14.51	17	9.83	0.152		
Chloroquine	17	6.67	17	9.83	0.235		
Amoxicillin	6	2.35	4	2.31	0.978		
Coartem	6	2.35	2	1.16	0.370		
Traditional medicine	1	0.39	2	1.16	0.353		
**Best place to go when a child has malaria**							
Hospital	195	76.47	112	64.74	0.008	0.56	(0.37–0.87)
Health Center	44	17.25	57	32.95	<0.001	2.36	(1.48–3.74)
Private doctor	13	5.10	1	0.58	0.010	0.11	(0.01–0.85)
Pharmacy	2	0.78	2	1.16	0.695		
Traditional healer	1	0.39	1	0.58	0.782		
**Have you received any advice related to malaria?**							
No	169	66.27	123	71.10			
Yes	86	33.73	50	28.90	0.293	0.80	(0.52–1.22)
**Sources of information regarding malaria**							
Hospital/Health Center	43	16.86	27	15.61	0.73	0.91	(0.54–1.54)
Family member	27	10.59	11	6.36	0.131	0.57	(0.28–1.19)
Neighbour	11	4.31	5	2.89	0.446	0.66	(0.22–1.94)
CHW	0	0.00	7	4.05	0.001	-	-
Radio	5	1.96	0	0.00	0.064	-	-
None	169	66.27	123	71.10	0.293	1.25	(0.82–1.91)

Asked about if they had received any advice related to malaria, only 33.73% of urban caregivers and 28.90% of rural reported to had received any information about malaria from one or more sources (Table 4). Of several interpersonal sources of information, most caregivers (16%) reported that they have received information from a hospital or health centre, followed by a family member (9%) or neighbour (4%). Only rural caregivers reported receiving information from community health workers, and only urban caregivers mentioned the radio as a source of information.

### Malaria Knowledge Score

**Fig 1:**
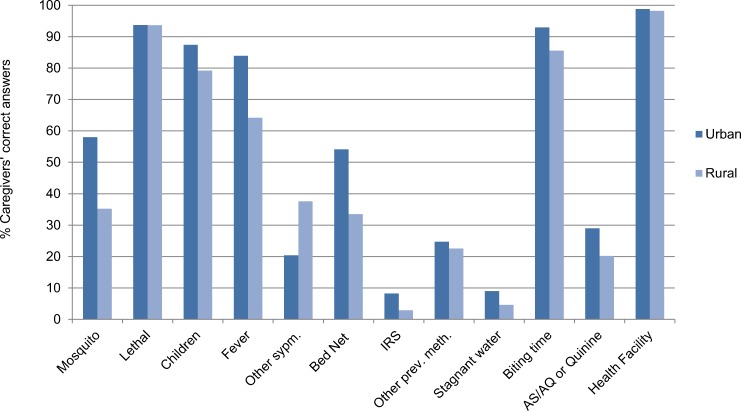
Caregivers’ malaria knowledge by area in Bata district, Equatorial Guinea. (Mosquito): Malaria is transmitted by a mosquito bite; (Lethal): Malaria can be lethal; (Children): Children suffer most frequently from malaria; (Fever): Fever is a malaria symptom; (Other symp.): Headache, body pains, or convulsions are malaria symptoms; (Bed Net): Sleep under a bed net prevents malaria; (IRS): Indoor spraying prevents malaria; (Other prev. meth.): Other correct prevention methods; (Stagnant water): Stagnant water is the breeding site of mosquitoes; (Biting time): Correct biting time of malaria mosquitoes; (AS/AQ or Quinine): First line malaria treatments; (Health facility): Place to look for treatment Health Centre, hospital or private doctor.

The majority of Bata district caregivers’ (58.41%) had a low Knowledge of Malaria Score with none of the 428 caregivers’ interviewed achieving the maximum score of 13 points (S2 Fig). The total median score was 6 with most of rural caregivers (69.94%) scoring Low Knowledge and half of urban caregivers (49.41%) scoring above the median (OR: 0.44 95% CI: 0.29–0.66). In general, rural caregivers’ malaria knowledge was lower than urban caregivers (Fig 1).

### Factors related with the knowledge of malaria

Caregivers with a secondary school education (at least), and belonging to the richest socioeconomic status had the highest odds of having a high knowledge of malaria score (Table 5). On the other hand, caregivers from houses with at least one case of malaria at the time of the survey had 0.65 times the odds of having a high malaria score than caregivers from houses without a malaria case (95% C.I. 0.42–0.99).

**Table 5:** Factors associated with Malaria Knowledge Score determined by multiple logistic regression.

	Unadjusted OR (95% CI)		Adjusted OR (95% CI)*	
**Age**				
15–24	1		1	
25–34	1.30	(0.76–2.22)	1.39	(0.78–2.48)
35–44	1.14	(0.62–2.10)	1.62	(0.80–3.25)
45–54	0.62	(0.32–1.21)	1.19	(0.54–2.64)
55 & +	0.46	(0.19–1.10)	0.71	(0.25–2.02)
**Education**				
Primary school or less	1		1	
Secondary school or more	2.88	(1.93–4.31)	2.34	(1.44–3.80)
**Wealth**				
Poorest	1		1	
Second	1.63	(0.85–3.12)	1.55	(0.73–3.32)
Middle	1.87	(0.97–3.62)	1.65	(0.72–3.76)
Fourth	2.29	(1.20–4.36)	2.10	(0.92–4.78)
Richest	5.60	(2.90–10.84)	4.31	(1.37–7.77)
**Malaria case in the house**				
No	1		1	
Yes	0.65	(0.42–0.99)	0.70	(0.44–1.12)

*Adjusted by area

After adjusting by area, the associations between malaria knowledge and caregivers’ level of education and wealth remained. Caregivers with secondary education had 2.34 times the odds of receiving a high malaria knowledge score compared to those with primary school education or less (95% C.I. 1.44–3.80). The association between wealth and the high Knowledge of Malaria showed a trend, with the richest households having 4.3 times the odds of receiving a high Knowledge of Malaria score compared to the poorest households (95% C.I. 1.37–7.77).

## Discussion

Adequate understanding of household knowledge about malaria transmission, prevention and treatment has proven to be crucial for the success of control programmes aimed at reducing childhood malaria morbidity and mortality [2,28]. This study provides relevant information about the extent of malaria knowledge, beliefs and attitudes in the Bata district of Equatorial Guinea. The caregivers from Bata were generally aware of malaria but their depth of knowledge was poor, especially in rural areas, where there is a higher prevalence of malaria. Furthermore, this study found that households with at least one child with malaria at home, households where the caregiver had an educational attainment of primary school level or less and households with a poor socioeconomic status were correlated with the low Knowledge of Malaria.

### Malaria awareness and symptoms

In the Bata District, malaria was reported to be the most important health problem, as in many other countries in Africa [2,4,5,29]. The comparatively higher awareness of this among rural caregivers could correlate with greater disease prevalence in those areas.

The recognition of two or more symptoms of malaria is essential for the in-home management of this disease among children [3]. Caregivers in Bata district were familiar with the main signs and symptoms associated with malaria, as are many other populations living in malaria endemic areas [4,5,27]. Although rural caregivers had 3 times lower odds of mentioning fever as a symptom, they had 4 times the odds of mentioning convulsions compared to urban caregivers. In Bata district, delay in seeking treatment for children with malaria is more frequently in rural households [22] and this delay could be responsible for the more frequent reports of convulsions in that area. However, the occurrence of convulsions in Bata district seems not to be linked to a prompt or different pattern in seeking treatment [19], suggesting that there is a need for more study about caregiver awareness of malaria convulsions and disease severity in children.

### Malaria transmission and prevention

Similarly to findings from other studies [30–32], malaria transmission and prevention was not well understood in Bata district, especially in rural area where households were less likely to mention that mosquitos transmit malaria. This gap in knowledge may have a significant impact on local prevention; to know that mosquitoes transmits malaria is crucial for the adequate use of the available malaria prevention tools at the household level [4,33,34].

In the Bata district, misconceptions about the role of mosquitos in malaria transmission were also evident in responses about prevention and the avoidance of mosquito bites. Most caregivers knew that malaria is preventable but “sleep under a bed” net was mentioned more frequently in the context of avoiding mosquito bites rather than malaria prevention, especially in rural areas. A poor appreciation of the protective effects of mosquito nets has been found elsewhere [3,35] with the avoidance of mosquito bites most frequently described as a reason to use mosquito nets [36,37]. This misconception could be one of the reasons why in Equatorial Guinea bed nets are more frequently used to protect adults than children [21].

Indoor residual spraying (IRS) was the principal activity implemented by the Malaria Control Programme in the region but it was infrequently mentioned by caregivers as a means of malaria prevention, especially those from rural areas. This approach was also more frequently described as a method of mosquito bite avoidance rather than prevention. Despite this, most caregivers in the Bata district said that they would permit government workers to spray their houses and these visits are a valuable opportunity to improve malaria household knowledge. Moreover, it seems that messages delivered by IRS campaign workers need to be reinforced if we are to improve the level of knowledge about malaria transmission in the district of Bata.

Whilst caregivers seemed to be aware of the need to avoid mosquito bites and when exactly these bites occurs, there were important misconceptions about mosquito breeding sites that could negatively influence household prevention. Most Bata caregivers believed that mosquito’s breed in garbage and bushes rather than in stagnant water. Consequently, substantial effort is expended on clearing bushes and grasses instead of draining stagnant water in or near the house [30,31,38].

### Malaria treatment, treatment sources and information

Any mention of AS+AQ or Quinine as first line treatments for children with malaria in Equatorial Guinea was very low, especially in rural areas. The commonest antimalarial mentioned by most caregivers was Artemether. The lack of adherence to national treatment guidelines and the preference for this artemisinin monotherapy in the Bata district has been already described [19]. It becomes necessary to educate households, and all actors involved in malaria treatment, about the risks linked with this long treatment [39]. Improving the quantity and quality of this message could have a large impact on children receiving effective ACTs [25].

As with other countries in the region [4,11], most of the caregivers in the Bata district affirmed that they would seek treatment for a child with malaria at a health care facility and hospitals were most frequently mentioned, with rural caregivers having twice the odds of visiting a health centre and urban caregivers had nine times the odds of visiting a private doctor. This might reflect the availability of facilities by areas, together with the capacity to finance private healthcare. It is remarkable that community health workers were not mentioned by households surveyed as treatment facilitators. This might be due to shortages in malaria treatment that have occurred in the Bata district since 2011.

In order to improve knowledge and promote effective prevention, it is important to use communication channels that are most likely to be used by local households. As commonly found in this region, Bata caregivers have received pertinent information about malaria from a health facility or family member more frequently, and less frequently from mass media [2,11,25]. Accordingly, the burden of health care provision falls on health facilities and workers, who should be supported in disseminating accurate and reliable information. However, the low number of households that had received any information or advice related to malaria in either area is a concern that should be addressed, especially for those households most at risk. Home education has been shown to be valuable in terms of malaria management [25] but in Bata district only few rural caregivers mentioned to have received information from the existing community health workers.

### Factors related to the Knowledge of Malaria

In the Bata district, the factors most significantly associated with the Knowledge of Malaria were rurality, level of education and socioeconomic status, factors also correlated with malaria knowledge in other studies in the region [40–42]. Usually, urban households receive more information due to their proximity to health facilities [26]. Furthermore, educated communities are also exposed to multiple sources of information in comparison to their more poorly educated counterparts [24,43]. While illiteracy excludes access to written information, health education may help caregivers to understand key aspects of the disease and improve their ability to assimilate information and explanations [27]. Increased caregiver knowledge about malaria may also promote continued interest in participating in control programs [2,44].

Inequalities in wealth could influence the provision of information about malaria among households [35]. An improved behavioural change communication strategy, based on the key elements of malaria control interventions, has been proven to help individuals and communities to overcome these inequalities in health provision [14,45].

To have at least one case of malaria at home at the time of the survey was associated with low Knowledge of Malaria in Bata district. Adequate knowledge of caregivers about malaria has been found to correlate with reduced morbidity and mortality among children [28].

This study has some limitations. First, this is a cross sectional study conducted in the Bata district alone, thus findings might not generalize to the whole country. Secondly, due to sample size, some associations may not show significance in multivariable logistic regression analyses, such as the presence of a malaria case in the house.

## Conclusions

Caregivers’ from the poorest wealth quintile, with a primary school level of education or less, and living in rural areas, had the lowest knowledge of malaria in the Bata District. Capacity now needs to be built in such households to empower them with adequate knowledge aimed at behavioural changes to improve disease prevention. The EGM Control Initiative should now update its health education strategy according to the identified gaps in caregiver’s knowledge, especially those regarding the role of the mosquito in transmission, together with the most efficacious forms of prevention and treatment for children. This could be achieved through communication strategies aimed at changing individual and community behaviours, and delivered by appropriately trained community health workers and IRS personnel, in a language considered to be most accessible and appropriate to vulnerable households.

## Supporting Information

S1 Fig Map of Bata district. (TIF)

S2 Fig Caregivers’ Malaria Knowledge Score achieved by area in Bata district, Equatorial Guinea. Kolmogorov-Smirnov test (p = 0.000).
(TIF)

S1 Questionnaire Knowledge, beliefs and attitudes questionnaire. (PDF)
