# Supplementary figures and images for: Caregivers’ Malaria Knowledge, Beliefs and Attitudes, and Related Factors in the Bata District, Equatorial Guinea
Source: PLoS One. 2016 Dec 30;11(12):e0168668. doi: 10.1371/journal.pone.0168668 (PMC5201263; doi:10.1371/journal.pone.0168668)

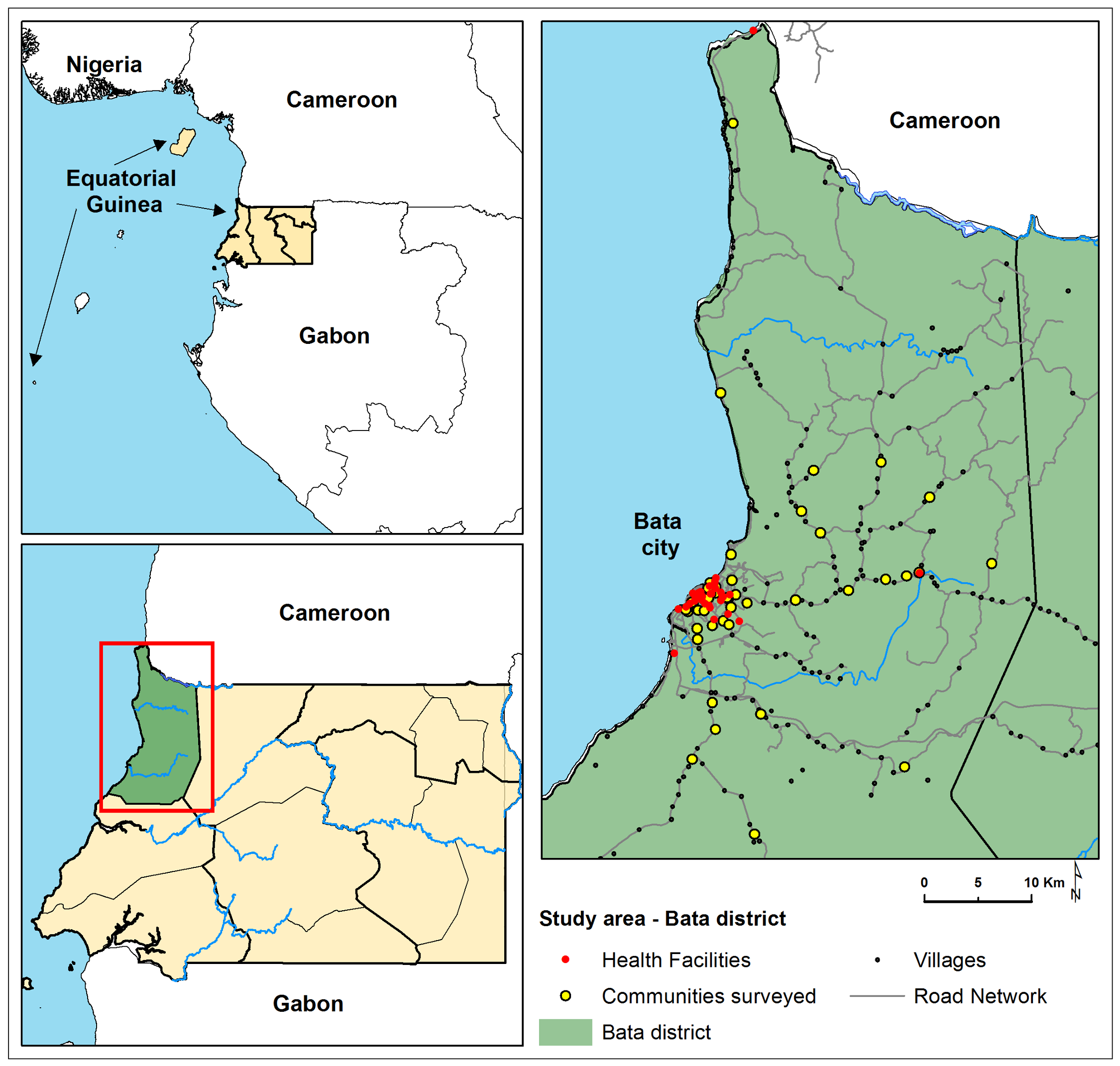

Supplement: S1 Fig — (TIF) [file pone.0168668.s001.tif]

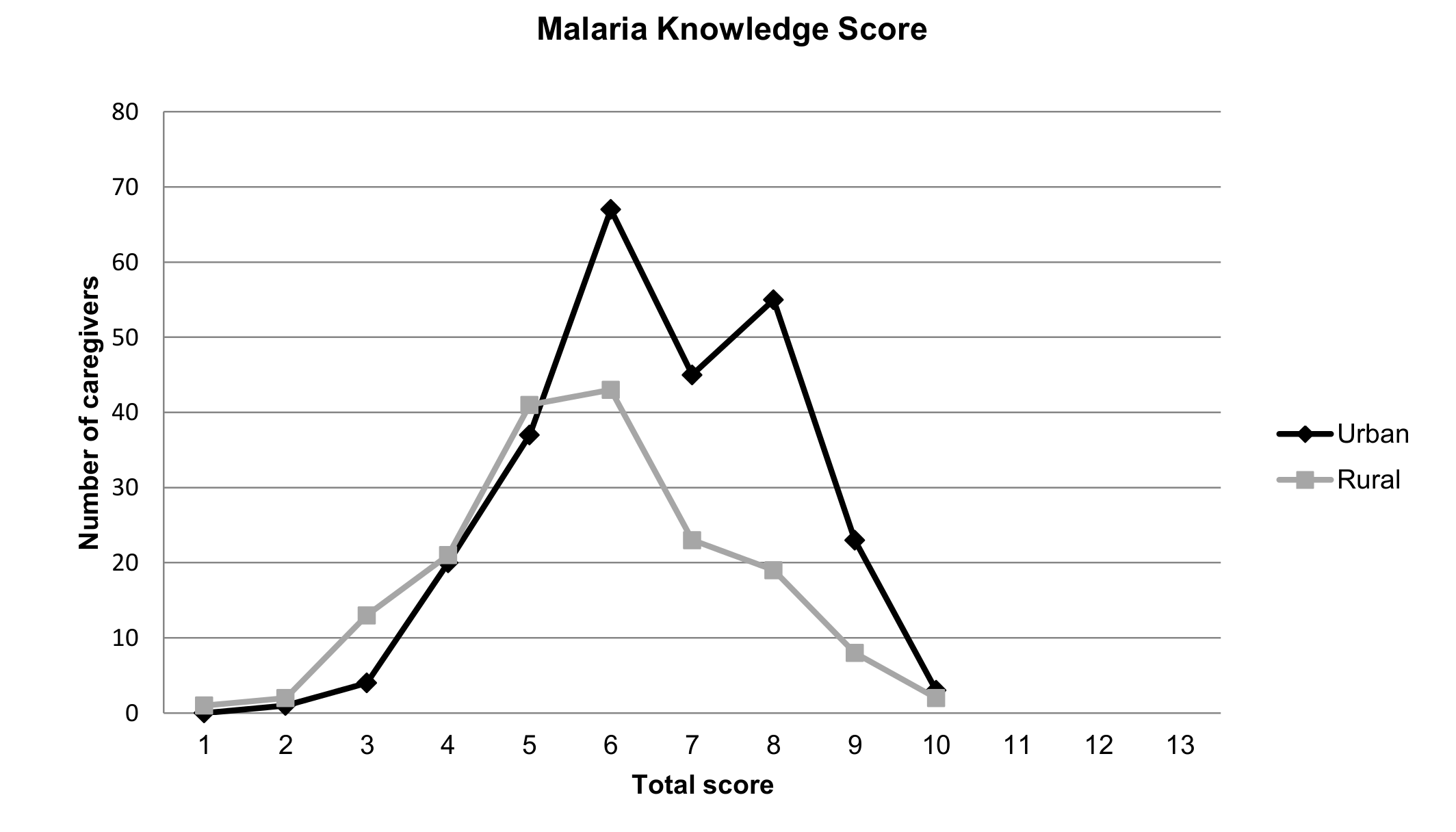

Supplement: S2 Fig — Kolmogorov-Smirnov test (p = 0.000). (TIF) [file pone.0168668.s002.tif]
