# Supplementary material for: Caregivers’ Malaria Knowledge, Beliefs and Attitudes, and Related Factors in the Bata District, Equatorial Guinea
Source: PLoS One. 2016 Dec 30;11(12):e0168668. doi: 10.1371/journal.pone.0168668 (PMC5201263; doi:10.1371/journal.pone.0168668)
Supplement: S1 Questionnaire — (PDF) [file pone.0168668.s003.pdf]

C. KNOWLEDGE, BELIEFS AND ATTITUDES.

Now we are going to talk about what you know and belief. Please, it is very important that you answer sincerely. There are no wrong answers; all your answers are good.

C.1.What is the principal health problem in your community?

1. Malaria
2. HIV/AIDS
3. Fever
4. Convulsions
5. Respiratory Infecctions
6. Diarrohea
7. Measels

98. Other ____
99. DK

C.2 Have you ever heard about malaria?

1. Yes
2. No

99. DK

C.3. Do you know any malaria symptom? (More than one answer is possible)

1. Fever
2. Convulsions
3. Body pain
4. Nausea
5. Headache
6. Weakness

98. Other ____
99. DK

C.4. How is malaria transmitted?

1. Mosquito bite
2. Dirtiness
3. Drinking water
4. Food
5. Walk barefoot

98. Other ____
99. DK

C.9. Who does suffer most frequently from malaria?

1. Children

2. Adults
3. Young
4. Children and young
5. Women
98. Other ____
99. DK

C.10. Who do you think malaria is more dangerous for?

1. Children
2. Adults
3. Young
4. Children and young
5. Women
98. Other ____
99. DK

C.11. Do you think malaria could be lethal?

1. Yes
2. No
99. DK

C.12. What are the main malaria symptoms in children? (More than one answer is possible)

1. Fever
2. Convulsions
3. Body pain
4. Nausea
5. Headache
6. Weakness
98. Other ____
99. DK

C.13. Do you know if malaria is preventable?

1. Yes
2. No (Go to C15)
99. Dk (Go to C15)

C.14. What is the best way to prevent malaria? (More than one answer is possible)

1. Indoor spraying
2. Generating smoke
3. Sleeping under a bed net
4. Cleaning the house and environs
5. Draining/covering stagnant water

6. Taking preventive medication
98. Other____
99. DK

C.15. And what is the best treatment for malaria? (More than one answer is possible)

1. Fansidar
2. Coartem
3. Chloroquine
4. Paracetamol
5. Amoxicillin
6. Quinine
7. Artemether
8. AS/AQ
9. Tradicional medicine
98. Other____
99. DK

C.16. Do you know the breeding site of mosquitoes?

1. Puddles
2. Grass
3. Dark places
4. Stagnant water
5. Garbages
98. Other____
99. DN

C.17. What is the biting time of mosquitoes that transmit malaria?

1. Dusk
2. Morning
3. Evening
4. Dawn
5. Night
98. Other____
99. DN

C.18. What is the best way to avoid a mosquito bite?

1. Use mosquito coil
2. Indoor spraying
3. Generate smoke
4. Sleep under a bed net

5. Clean the house and environs
6. Nets in the windows
98. Other ____
99. DN

C.19. If a child has malaria, where is the best place to seek treatment?

1. Pharmacy
2. Traditional healer
3. Private doctor
4. Heath Center
5. Hospital
98. Other____
99. DK

C.20. What is the best treatment for a child with malaria?

1. Fansidar
2. Coartem
3. Chloroquine
4. Paracetamol
5. Amoxicillin
6. Quinine
7. Artemether
8. AS/AQ
9. Tradicional medicine
98. Other____
99. DK

C.21 Have you ever received any advice related to malaria?

1. Yes (Go to 22)
2. No
99. DK

C.22. Where or from who have you received this advice?

1. Mother
2. Radio
3. Hospital
4. Health Center
98. Other____
99. DK
